# Standardizing post-cardiac arrest care across rural–urban settings – qualitative findings on proposed post-cardiac arrest learning community intervention

**DOI:** 10.1186/s12913-023-10147-w

**Published:** 2023-11-15

**Authors:** Teresa L May, Skye Siladi, Alison L Daley, Richard Riker, Rita Zanichkowsky, Michael Burla, Erica Swan, Jean A Talbot

**Affiliations:** 1 Department of Critical Care, Maine Medical Center, Portland, ME USA; 2 Acute Care Center of Biomedical Research Excellence, Portland, ME USA; 3 Muskie School of Public Service, University of Southern Maine, Portland, ME USA; 4 Department of Emergency Medicine, Southern Maine Medical Center, Biddeford, ME USA; 5 MaineHealth Corporate, Portland, ME USA

**Keywords:** Cardiac arrest, Implementation science, Rural health

## Abstract

**Background:**

Standardization of post-cardiac arrest care between emergency department arrival and intensive care unit admission can be challenging, particularly for rural centers, which can experience significant delays in interfacility transfer. One approach to addressing this issue is to form a post-cardiac arrest learning community (P-CALC) consisting of emergency department (ED) and intensive care unit (ICU) physicians and nurses who use data, shared resources, and collaboration to improve post-cardiac arrest care. MaineHealth, the largest regional health system in Maine, launched its P-CALC in 2022.

**Objective:**

To explore P-CALC participants’ perspectives on current post-cardiac arrest care, attitudes toward implementing a P-CALC intervention, perceived barriers and facilitators to intervention implementation, and implementation strategies.

**Methods:**

We conducted semi-structured, individual, qualitative interviews with 16 staff from seven system EDs spanning the rural–urban spectrum. Directed content analysis was used to discern key themes in transcribed interviews.

**Results:**

Participants highlighted site- and system-level factors influencing current post-cardiac arrest care. They expressed both positive attitudes and concerns about the P-CALC intervention. Multiple facilitators and barriers were identified in regard to the intervention implementation. Five proposed implementation strategies emerged as important factors to move the intervention forward.

**Conclusions:**

Implementation of a P-CALC intervention to effect system-wide improvements in post-cardiac arrest care is complex. Understanding providers’ perspectives on current care practices, feasibility of quality improvement, and potential intervention impacts is essential for program development.

**Supplementary Information:**

The online version contains supplementary material available at 10.1186/s12913-023-10147-w.

## Background

Patients with out-of-hospital-cardiac arrest (OHCA) have a high mortality and morbidity across the United States, with a 24% survival to hospital admission, 9% survival to hospital discharge and a 7% incidence of discharge with a good neurological status [1]. Globally, good outcomes after OHCA are known to be lower in rural areas compared to urban areas [2–5]. Although this difference has been attributed in part to prehospital variables, including availability of public defibrillators, incidence of bystander cardiopulmonary resuscitation (CPR), and longer rural emergency medical services (EMS) response and transport times, the quality of in-hospital care and volume of cases is also associated with outcomes [1, 2]. Whether survivors will be admitted to their initial hospital or require interfacility transfer to a tertiary hospital, Emergency Department (ED) providers must be prepared to deliver post-resuscitation-specific care in all situations. For EDs in rural areas, where the volume of patients presenting after cardiac arrest is low, providing specific care can be challenging.

Optimizing post-resuscitation care for patients in the rural setting is of particular concern in states like Maine, where more than 60% of residents live in rural areas with rural–urban healthcare disparities identified for several diseases [6–8]. MaineHealth, the largest regional health system in the state, developed a Post-Cardiac Arrest Learning Community (P-CALC) to standardize care following return of spontaneous circulation (ROSC) in its EDs, and strengthen the relationship between rural EDs and tertiary intensive care unit (ICU) teams. This qualitative study interviewed participants to better understand their perspectives on the barriers and facilitators associated with the current approach to post-cardiac arrest care in EDs and on the potential practice impacts of a P-CALC intervention. The process of the P-CALC can be found in Supplement 1 Fig. 1.

## Methods

### Forming the post-cardiac arrest learning community

The P-CALC members consist of a physician and nurse champion at each of 9 EDs and 2 ICUs that routinely manage post-cardiac arrest patients within the MaineHealth system. Other participants include data analysts, community consultants, cardiology consultants, research coordinators and qualitative consultants. The approach to P-CALC is multifaceted, which includes team building, protocol and resource sharing, data review and collaborative quality improvement. Yearly, the P-CALC leadership conducts site visits of each of the EDs to learn about the facilities and better understand both global and cardiac-arrest specific challenges each center faces. A post-cardiac arrest protocol specific for the ED-to-ICU time period was developed and collaboratively reviewed. Then an order set was built into the electronic heath record that is shared among all centers. An electronic dashboard was created to provide P-CALC sites with performance data including patient-level arrest data, information on each post-cardiac arrest patient’s arrest, blood pressure, oxygenation, ventilation, temperature and glucose values, and time-stamped data describing pressor medications, ventilator changes, and insulin dosing in the ED and the ICU. The dashboard also lists times patients spend in the ED, during intra-facility transfer, in the cardiac catheterization lab, and in the ICU. Monthly P-CALC meetings are conducted to review cases, share evidence of best practices, invite cardiac arrest survivors and their families to describe their experiences and speak with their care team, discuss strategies for standardizing hyperacute post-cardiac arrest management, and identify site-specific barriers and facilitators to patient management.

From the P-CALC members, we recruited individuals to participate in structured interviews [including author MB]. We used purposive sampling to recruit ED staff representing different types of sites (rural, regional, and tertiary care center EDs) and roles (physician and nurse) [9]. These staff were contacted via email, provided with information about the study, and invited to join. Before their interviews, participants were provided with a handout reviewing the study purpose and data management plan. This study was evaluated by the MaineHealth Institutional Review Board and was granted exemption from requiring ethics approval, according to federal regulations.

### Conceptual framework

This study was informed by the Consolidated Framework for Implementation Research (CFIR). Synthesizing multiple evidence-based theories, the CFIR identifies and organizes concepts shown to be important in influencing the implementation of health services innovations. CFIR constructs describe several domains relevant to implementation including characteristics of health systems and sites within these systems, innovation characteristics, and implementation processes [10]. We used CFIR constructs to guide our selection of broad research topics and to inform our development of interview questions, coding categories, and themes.

### Interview guide development

The research team produced a semi-structured interview guide designed to elicit participants’ perspectives on current post-cardiac arrest care at their EDs; the value of the proposed innovation; implementation barriers and facilitators; and potentially effective implementation strategies (Supplement 1). The interview guide was developed with input from a board-certified critical care specialist, a P-CALC facilitator with extensive experience in healthcare quality improvement (QI), and two investigators with qualitative research expertise. To verify the face validity, comprehensibility, and appropriateness of questions, the interview guide was pilot tested with an experienced ED physician who was not a study participant.

### Data collection

From October 2021 to January 2022, participants were interviewed individually by the two qualitative researchers, *who had no prior relationships with participants and who were not involved in any other aspects of P-CALC. To limit the effects of social desirability bias on participants’ responses, the Principal Investigator did not take part in data collection. The P-CALC facilitator attended interviews to observe, ask clarifying questions, and prepare field notes. Although the presence of this researcher may have introduced potential for social desirability bias, the team decided that the benefits of her participation outweighed any disadvantages, as her deep subject matter expertise helped to ensure that participants’ responses were accurately understood and fully explored.*

To enhance interpretive validity, participants were given opportunities to elaborate on topics and introduce themes of interest to them, with probes used as needed to ensure that participants’ meanings were adequately captured [11]. Code saturation was achieved by the fourteenth interview: the addition of the last two interviews increased the number of new codes by only 1.5% relative to the number of codes in the base (Guest 2020). Interviews ranged from 30 to 60 min in length and were conducted virtually from private locations. Interviews were audio-recorded, transcribed verbatim, and uploaded into NVivo © qualitative analytic software.

### Analysis

In analyzing interview data, we used directed content analysis [12] within a team approach [13]. First, a preliminary codebook was developed including codes based on CFIR constructs and sub-codes derived inductively from the transcribed data [13, 14]. The preliminary codes were reviewed by the team and the codebook was edited to reflect team recommendations.

Using this revised codebook, two researchers independently coded an interview transcript, then compared their coding, clarified concepts, and edited the codebook again to reach consensus on the definition and use of codes. These two researchers conducted three additional rounds of coding and review and to further refine the codebook. A single researcher applied the finalized codes to the remaining transcripts and identified themes in the coded data. These themes were reviewed by the team and revised in response to team feedback.

## Results

The interviews represented seven EDs (five rural, one regional, and one tertiary) within the same health system. Of the 19 individuals invited, 16 (eight physicians and eight nurses) contributed interviews. Nine participants identified as female and seven as male. Nine were based at rural centers, three were from the regional center, and four were from the tertiary care center.

### Facilitators and barriers to current post-cardiac arrest care in EDs

In discussing facilitators and barriers to their current provision of post-cardiac arrest care, participants identified factors at the site and system levels. Table 1 summarizes relevant themes. See Supplement Table 2.1 for additional exemplary quotes.

**Table 1:** Facilitators and barriers to current provision of post-cardiac arrest care in system EDs

Topic	Facilitator vs. Barrier	Themes from P-CALC Data
Site-Level Factors	Facilitator	1.1: Effective communication was cited as a strength for some ED teams
	Facilitator	1.2: Teams showed strengths in provision of hyper-acute care
	Barrier	1.3: Some ED staff felt less well prepared to manage post-cardiac arrest patients for longer periods
	Facilitator	1.4: Staff-to-patient ratios were seen as adequate at some sites
	Barrier	1.5: Staff shortages could interfere with post-cardiac arrest management
System-Level Factors	Barrier	1.6: Limited bed availability at receiving hospitals posed problems for EDs’ provision of optimal post-cardiac arrest care
	Barrier	1.7: Rural ED leadership reported challenges negotiating administrative process connected with post-cardiac arrest patient transfers to tertiary care centers
	Barrier	1.8: Rural EDs reported difficulties in arranging for EMS to transport post-cardiac arrest patients to receiving hospitals
	Barrier	1.9: Tertiary care center reported receiving incomplete histories on post-cardiac arrest patients transferred from EDs at smaller facilities

#### Site-level factors

Nurses highlighted their teams’ strengths in effective, respectful communication as a factor enhancing the quality of the post-cardiac arrest care they provided (Theme 1.1):

[W]here we are such a small hospital, we're a tight-knit community. We run such smooth codes because we're all good at…establishing our roles, knowing who's in charge. And then we're really…pushing for that closed-loop communication, and it's been phenomenal (Rural Nurse #2).

In discussing their teams’ mastery of skills needed to care for post-cardiac arrest patients, physicians and nurses saw ED staff at their sites as expert in hyper-acute care (Theme 1.2). However, participants indicated that ED staff sometimes felt less well equipped to manage these patients for long periods beyond their initial stabilization (Theme 1.3). As a tertiary care center Nurse #2 commented: “We stabilize, we're good at that. And then that next step of care, we don't have a lot of practice with.”

Although two physicians—one at a rural hospital and one at the tertiary care center—saw their staff-to-patient ratios as acceptable (Theme 1.4), other nurses and physicians described them as suboptimal. Some noted that this problem was intensified by the COVID-19 pandemic, which increased the volume of critical care patients in EDs. Staffing shortfalls were seen as potentially interfering with management of post-cardiac arrest patients (Theme 1.5).

#### System-level factors

Participants across all site types identified a lack of bed availability at receiving hospitals—including the system’s tertiary care center—as a major obstacle in providing optimal post-cardiac arrest care (Theme 1.6) The COVID-19 pandemic was seen as exacerbating these shortages. However, two rural physicians recalled such problems before the pandemic. Participants expressed concern that the lack of beds contributed to delays in transferring patients from EDs to definitive care sites: Rural Physician #5 observed that “[t]he boarding that we're doing [with post-cardiac arrest patients] is beyond any scope of boarding we've ever seen in the past, and certainly that's…one of the big challenges.”

Transfer delays could be particularly problematic for sites where staff were unaccustomed to providing post-cardiac arrest care beyond stabilization. Describing his younger physician colleagues’ response to this situation, Rural Physician #6 said, “[T]hey're having to manage people for a lot longer than they originally planned, and I think that's scary for a lot of people.” This respondent reported that his nursing staff also “struggled” to adjust to caring for critically ill patients over long periods, and said he carefully prioritized tasks he assigned to them, “because if I order too many things, it’s going to really overwhelm them.”

In addition to identifying problems related to bed availability, some rural physicians mentioned challenges associated with the administrative process surrounding transfers of post-cardiac arrest patients to receiving hospitals, including their own system’s tertiary care center (Theme 1.7). The process was described as cumbersome, lacking standardization, and typically requiring multiple phone calls to various specialists at accepting institutions:

I've had issues where… I have a full ER and I'm dealing with a cardiac arrest and …when I'm making a phone call and getting on the line… [T]hat uses up some of my time and energy when I would rather be taking care of the patient at that moment (Rural Physician #6).

Participants from three rural sites reported challenges arranging EMS transport for post-cardiac arrest patients to definitive care (Theme 1.8). Coordinating ground transport was described as “a huge hurdle” (Rural Nurse #3) in rural areas because local EMS services were often fully occupied providing 911 service to their communities, and they lacked the vehicles, staff, and time needed to deliver patients to distant receiving hospitals. One rural physician reported that he often waited four to six hours for EMS to arrive and begin the 90-min trip from his site to the system’s tertiary care center.

In describing their experience receiving patients transferred from smaller EDs, staff at the system’s tertiary care center indicated that important details might sometimes be missing from the histories they obtained from EMS crews responsible for transport (Theme 1.9).

### Attitudes toward the P-CALC intervention

In discussing their attitudes toward the intervention, participants evaluated its underlying principles and goodness-of-fit to site-level challenges. They also considered the relative importance of site-level and system-level factors in affecting post-cardiac arrest outcomes and appraised proposed plans to distribute performance data to participating sites. See Table 2 for a list of themes and Supplement Table 2.2 for additional exemplary quotes.

**Table 2:** Participant attitudes toward the P-CALC intervention

Topic	Expressed Approval vs. Concern	Themes from P-CALC Data
Intervention Goals and Principles	Approval	2.1: Participants voiced support for initiatives to improve post-cardiac arrest care in EDs
	Approval	2.2: Participants expressed approval for specific aspects of P-CALC intervention
	Concern	2.3: Participants raised questions or concerns about aspects of the intervention rationale
Intervention Goodness-of-Fit	Concern	2.4: ED services may need to be expanded because post-cardiac arrest patients now board in EDs for longer periods
	Approval	2.5: System EDs need to standardize post-cardiac arrest care in order to optimize quality
	Approval	2.6: Standardization of post-cardiac arrest care could help decrease stress associated with addressing a low-frequency, high-acuity event:
	Approval	2.7: Focus on intervention targets might impede staff’s efforts to prepare post-cardiac arrest patients for transfer
Importance of Site-Level vs. System-Level Factors	Concern	2.8: To achieve optimal post-cardiac arrest care, QI within EDs must be paired with system-level efforts to streamline transfers
	Concern	2.9: QI within EDs is necessary but not sufficient and should be accompanied by standardization of communications related to transfers of post-cardiac arrest patients from EDs to tertiary care centers
Dissemination of Performance Data	Approval	2.10: Dissemination of system-wide and site-specific performance data on intervention targets could help support staff engagement
	Concern	2.11: Physicians expressed reservations about distribution of performance data

#### Intervention goals and principles

All participants expressed support for QI efforts targeting post-cardiac arrest care in system EDs (Theme 2.1), embracing such ED initiatives that “pave[d] the way for the patient’s success or failure” (tertiary care center physician), with several physicians noting that appropriate interventions in the acute phase were necessary to preserve brain functioning: “[T]he mantra for stroke, ‘Time is brain,’ I think applies here” (Regional Physician).

Participants also endorsed specific aspects of the approach proposed in this project (Theme 2.2). First, some physicians appreciated the project’s objective of ensuring that both rural and urban EDs were able to provide high-quality post-cardiac arrest care. Rural Physician #6 commented, “[M]y whole philosophy is that someone in a rural area shouldn’t get substandard care…so I think we should be able to rise to the occasion.” They also valued P-CALC’s emphasis on standardizing post-cardiac arrest ED care and agreed that increased attention to the six clinical targets selected for the intervention would likely improve patient outcomes.

Although all participants saw the intervention as potentially beneficial, a subset representing all site types had questions or concerns about the rationale underlying intervention components (Theme 2.3). Some asked whether there was evidence supporting selected targets for glucose, temperature, or use of paralytics. Physicians at four sites suggested that it might be important for them to use their clinical judgment in deciding how strictly to adhere to aspects of the protocol or how to prioritize its targets. Some suggested that modification of targets might sometimes be necessary to avoid harm to patients.

#### Intervention goodness-of-fit

Participants said they believed the intervention would help them cope with challenges they faced at their sites. Some indicated that because the lengths of post-cardiac arrest patients’ ED stays had recently increased, ED staff might need to engage in QI to broaden the scope of services provided before their transfer (Theme 2.4).

Participants identified two reasons why sites would benefit from greater standardization of post-cardiac arrest care. First, their current processes of care were inconsistent, and eliminating this variability through implementation of evidence-based protocols could enhance quality (Theme 2.5):

[I]f we’re not using appropriate techniques and protocols for those patients… they end up ultimately passing at our facility… [I]t’s sometimes frustrating to know that we’ve done what we know to do and it hasn’t been enough. But maybe if we had a set protocol to follow, we could do better (Rural Nurse #1).

Second, some physicians observed that high-acuity post-cardiac arrest patients were seen infrequently at their sites, and that staff had few opportunities to learn best practices. Under those circumstances, protocolization could provide necessary guidance and alleviate anxiety (Theme 2.6):

It’s such a severe illness, but it’s one that we don't see all the time. So it sometimes makes people nervous and not necessarily stay as calm or…as thoughtful as they can be. So…having this protocol for the nurses will actually be hugely reassuring (Rural Physician #5).

On the other hand, certain participants at rural sites with limited resources worried that attempts to meet intervention targets might compete with staff’s efforts to prepare patients for transfer and might therefore result in delays and adverse impacts (Theme 2.7).

#### Importance of site-level vs. system-level factors

Several participants observed that to optimize outcomes for post-cardiac arrest patients, QI within EDs must be accompanied by system-wide improvements to mitigate barriers to timely transfer (Theme 2.8):

Early post-resuscitation care lives in the ED. I don’t think we can argue that. But equally [important] is getting those folks out of the Emergency Department and into an ICU where they’ve got one-on-one nursing…I think we need to do both…[I]f we do one and not the other…we’re missing the mark. (Rural Physician #1)

Staff at the system’s tertiary care center also emphasized the need to protocolize communications surrounding transfers of post-cardiac arrest patients to their site from other system sites (Theme 2.9).

#### Dissemination of performance data

As stated above, one strategy of the proposed intervention is to provide participating sites with easily accessible system-wide and site-specific quality data on post-cardiac arrest care processes and outcomes. Participants across all sites endorsed this approach (Theme 2.10). Some noted that data could help ED staff appreciate that “what [they] did mattered” (Tertiary Care Center Physician); this insight, in turn, could serve as “a motivating factor” (Rural Physician #2, Tertiary Care Center Nurse #2) supporting staff’s adherence to high standards of care. Moreover, participants emphasized that sites would benefit from being able to compare their performance to that of other sites involved in the intervention; these cross-site comparisons could heighten staff’s awareness of system-level benchmarks, stimulate healthy competition, and help participating facilities identify successful strategies that might be disseminated across sites.

On the other hand, some rural physicians identified caveats associated with sharing performance data (Theme 2.11). For example, one cautioned that dissemination of data might discourage staff and compromise engagement in the intervention if ‘[w]e’re giving negative feedback to providers for things that are…in many ways out of their control” (Rural Physician #1).

### Facilitators and barriers to intervention implementation

In reflecting on P-CALC’s feasibility, participants described facilitators and barriers relating to characteristics of the intervention as well as to site- and system-level factors (Table 3). (See Supplement Table 2.3 for additional supporting data.)

**Table 3:** Facilitators and barriers to intervention implementation

Topic	Facilitator vs. Barrier	Themes from P-CALC Data
Intervention Characteristics	Facilitator	3.1: Intervention targets were seen as readily achievable
	Barrier	3.2: Intervention focused on QI for post-cardiac arrest care is inherently challenging because cardiac arrest patients are seen infrequently and opportunities to reinforce new practices are therefore rare
Site-Level Factors	Facilitator	3.3: Proposed intervention is feasible because it is compatible with ED’s current practice
	Barrier	3.4: Low staff-to-patient ratios could result in competing demands that interfere with post-cardiac arrest focused QI in EDs,
	Barrier	3.5: Acute stressors like the COVID-19 pandemic could limit staff’s availability to take part in intervention implementation
	Barrier	3.6: Infrastructure limitations at rural EDs might make some intervention targets more difficult to reach
	Barrier	3.7: Staff might need new skills training to implement intervention effectively
	Barrier	3.8: Sites may encounter difficulties in making sure that travel nurses receive the same intervention-related training as permanent staff
System-Level Factors	Facilitator	3.9: Health system has a successful track record of implementing cross-site QI mechanisms

#### Intervention characteristics

All physicians and four nurses from rural, regional, and tertiary care sites regarded intervention targets as readily attainable (Theme 3.1). However, some observed that QI efforts highlighting post-cardiac arrest care were inherently challenging because patients in this population presented to system EDs relatively rarely; thus, opportunities to practice the new skills and standards comprised by the intervention would be limited (Theme 3.2).

#### Site-level factors

In considering the extent to which the climate within their sites was conducive to intervention implementation, three physicians and five nurses across all site types observed that their teams’ current management of post-cardiac arrest patients was consistent with many of the intervention targets (Theme 3.3). However, low staff-to-patient ratios were identified as a challenge to current post-cardiac arrest care and regarded as potentially significant obstacles to post-cardiac-focused QI. Participants across all site types predicted that ED staff might struggle to learn and adhere to new protocols while coping with heavy competing care demands (Theme 3.4).

Several participants in rural and tertiary care sites identified pandemic-related burdens as a factor that could limit staff’s ability to commit to intervention implementation (Theme 3.5). Moreover, some participants cautioned that specific targets might be more challenging or even impossible to attain because of infrastructure constraints at their rural EDs (Theme 3.6).

Staff preparedness levels were cited as another potential barrier since some intervention components might be unfamiliar and new skills training might need to be provided to enable implementation (Theme 3.7):

[Staff will need training,] especially in use of [arterial] lines to manage blood pressure…I think there will be a lot of education that needs to happen in terms of certain medications that we're using (Rural Nurse #1.)

Relatedly, two participants—one from a rural and one from a regional ED—noted that their centers relied heavily on travel nurses to cope with workforce shortages, and they anticipated challenges in ensuring that these travelers received the same intervention-relevant training as permanent staff (Theme 3.8).

#### System-level factors

All five rural physicians observed that although system-wide standards for post-cardiac arrest care were not yet in place, their health system had successfully facilitated cross-site QI cooperation on other issues, including stroke, sepsis, trauma, and most notably, ST-segment elevation myocardial infarction (STEMI) (Theme 3.9). Participants described the system’s STEMI collaborative as having “robust” mechanisms (Rural Physician #1) for collecting quality metrics and sharing them with member sites to assist them in monitoring their adherence to best practices in STEMI care:

I would point to STEMI as …the gold standard within the institution, in terms of being able to have a program that’s very well-run, and the follow-up and the feedback is delivered in a regular and timely fashion (Rural Physician #3).

### Implementation plans

Participants described five strategies that they planned to use to raise staff awareness of the intervention and support implementation (Table 4). In addition to formal didactics and skills training (Strategy 1), where participants suggested using multiple communication techniques to ensure all staff were aware of the intervention (Strategy 2) such as frequent huddles and emails; development and dissemination of a formal, written practice standard; prominently posted visual displays listing intervention targets; and post-cardiac arrest specific order sets embedded in the electronic health record. Participants from rural and regional sites also noted their need to acquire appropriate equipment (Strategy 3), and they outlined plans for collecting and sharing site-specific data with their own teams (Strategy 4) as a means to develop more focused QI targets and support skills acquisition. Finally, some rural and regional participants stressed the importance of giving their teams “the opportunity to vet the process and the protocol and the guideline” (Rural Physician 1) (Strategy 5).

**Table 4:** Proposed implementation strategies

1. Conduct didactics and skills training
2. Develop communication plans to ensure all staff are aware of the intervention
3. Acquire necessary equipment
4. Collect and share site-specific performance data
5. Elicit and incorporate teams’ input into intervention plan

## Discussion

In this qualitative study on a proposed intervention to standardize post-resuscitation care for cardiac arrest patients across transferring EDs and receiving ICUs, we identified important themes in participants' perspectives on the current system of care, attitudes toward the intervention, expected facilitators and barriers to intervention implementation, and proposed implementation strategies. Providing care for patients following cardiac arrest in a rural ED is challenging for many reasons related to center volumes, infrastructure and resources. The sites identified existing strengths including effective communication within their teams, flexibility to adapt to new treatments, and expertise in stabilization of post-cardiac arrest patients. Challenges included the need to care for these patients for prolonged periods, low volumes of post-cardiac arrest patients in rural EDs, and barriers to timely transfer of patients from rural EDs to definitive care sites. Although rural centers welcomed new protocols and resources to optimize care for patients following cardiac arrest, the stress of delayed transfers, the COVID pandemic, and temporary staff were identified as barriers to be addressed.

Many studies have explored the effect of rurality on OHCA outcomes, but few have developed interventions to improve the quality of post-cardiac arrest care in rural EDs other than expediting transfer to regionalized centers [15, 16]. Our findings are important for this P-CALC framework and for other projects aiming to standardize acute care across rural and urban centers for this patient population. We found that the specifics of the intervention require not only a deep understanding of the system that is caring for these patients but also how challenges change over time, which highlights the importance of qualitative work within this type of intervention.

One major theme that emerged as an enduring issue was delayed transfer times, which were exacerbated by the COVID-19 pandemic, where increased boarding times were commonly seen [17]. It has been demonstrated that boarding patients in the ED has negative impact on patient outcomes, including OHCA patients [18]. The P-CALC was planned before the pandemic began, and although bed availability was not a planned topic in the P-CALC framework, these interviews clarified the need for a response to this issue. In light of this finding, the P-CALC participants have explored approaches for improving patient flow between hospitals, including ED-to-ED transfer when ICU bed availability is limited. The P-CALC team has also focused on standardizing interfacility transfer care, given the long drive times and frequent interruptions in helicopter services due to weather. There is ongoing engagement with local EMS services with the P-CALC team, as well as other care team members involved in transfers including respiratory therapists. Subsequent interviews and process metrics will be required to determine if these interventions are successful.

These findings highlight that providers at rural EDs are highly motivated to optimize the care they provide to OHCA patients, believing that patients in both rural and urban areas should receive high-quality care after cardiac arrest. Providers acknowledged that there was room to improve their care, and that this patient population could benefit from incorporating evidence-based principles and standardization into QI efforts. Providers enthusiastically agreed on the need to enhance collaboration between EDs and receiving centers. Members also endorsed the use of performance data as a motivating factor to adhere to high standards of care, although there was some concern expressed about receiving negative feedback from data reviews.

An inherent tension was identified between keeping patients in the ED to optimize their status before transfer versus the need to transfer as rapidly as possible. Participants considered MaineHealth’s existing stroke and STEMI programs as models to emulate, which rely on minimizing delays to cerebral or cardiac angiography, providing feedback to EDs and sharing quality metrics. Unlike patients with stroke and STEMI, patients with cardiac arrest do not receive a discrete intervention, but do require advanced treatment and monitoring, which can be initiated in the ED. Given the perspectives of our P-CALC participants, we plan a multi-targeted approach to improve transfer times and optimize care while awaiting transport.

This study is subject to several limitations. Although our sample was appropriate for study purposes [19], the size was small, and findings may not be generalizable to centers with different characteristics and circumstances. In particular, as this study emphasized experiences of rural EDs, viewpoints that might emerge in urban settings may be underrepresented in our findings. Interviews were only conducted with physicians and nurses most-directly impacted by the post-cardiac arrest protocol, therefore excluding possible valuable perspectives of other care team members. Selection bias may also have had an impact on results: Participants had all voluntarily agreed to take part in P-CALC. Thus, it can be assumed that they showed strong interest in cardiac arrest care and quality improvement and were therefore more likely to favor the intervention than similarly situated professionals who chose not to participate. Social desirability may have played a role as well: *Participants’ desire to maintain good relationships with colleagues on the research team may have led them to provide relatively positive evaluations of PCALC*. Finally, responses may have been affected by the point in time when data were collected. Participants’ views on barriers to provision of post-OHCA care were likely influenced by their recent experiences with the COVID-19 pandemic. In addition, interviews were conducted early in the life of P-CALC, and participant attitudes may change as the project evolves. Despite these limitations, insights gleaned from this study will likely help to inform quality improvement efforts in our system and in other health systems with rural reach.

## Conclusion

Identifying specific facilitators and barriers to standardizing post-cardiac arrest care across rural and urban centers is an important prerequisite as this P-CALC project is introduced. Our findings will help us address the concerns of our participants and gain from their strengths and strong enthusiasm for developing system-wide collaborations. Additional work is needed to determine how these findings evolve over time and whether the P-CALC program will improve adherence to best practices and result in improved outcomes for cardiac arrest survivors.

### Supplementary Information

**Additional file 1. Supplement 1.**

**Additional file 2:** **Supplement 2.** Themes and Additional Example Quotes.

## Data Availability

The interview guide is provided in the supplement. The codebook created during the study will be made available from the corresponding author on reasonable request.
