# Supplementary material for: Standardizing post-cardiac arrest care across rural–urban settings – qualitative findings on proposed post-cardiac arrest learning community intervention
Source: BMC Health Serv Res. 2023 Nov 15;23:1258. doi: 10.1186/s12913-023-10147-w (PMC10652430; doi:10.1186/s12913-023-10147-w)
Supplement: Supplementary file 1 — Additional file 1. Supplement 1. [file 12913_2023_10147_MOESM1_ESM.docx]

**SUPPLEMENT 1:**

**Supplement figure1: Process of P-CALC**


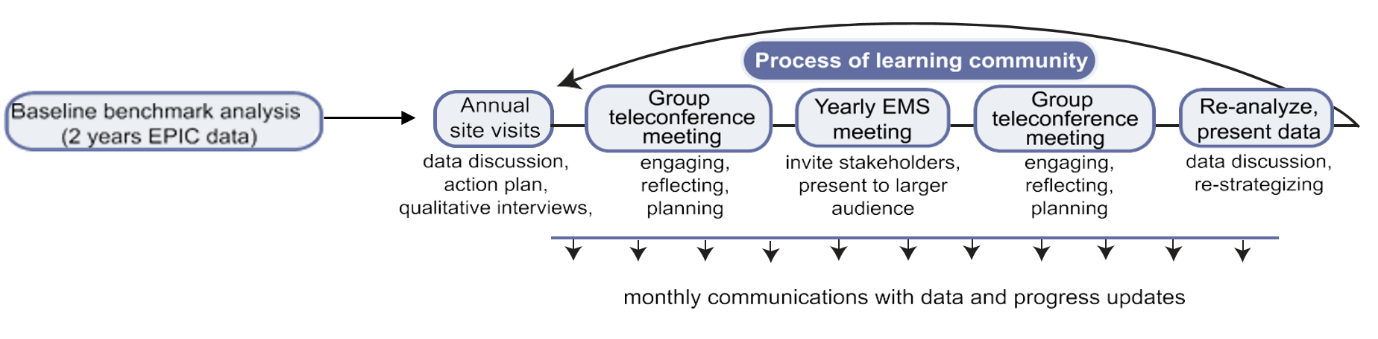


**INTERVIEW GUIDE**

**Current Processes of Care at Key Informant’s Site**

- At your center, how do different services work together to care for cardiac arrest patients?
  - *1(1a)* What aspects of care are well coordinated across services?
  - *1 (1b)* What are some challenges that come up in coordinating care for these patients across services?
- To what extent does your center typically collaborate with other MaineHealth organizations to work on quality improvement?

**Intervention Goodness-of-Fit**

In the scope of care for cardiac arrest patients, how much do you think management in the ED affects long-term outcomes?

- Do you think there is a need for improved post-cardiac arrest care at your center?
- At the initial site visit, you received an overview of the project rationale that explained why specific therapeutic targets were chosen as the focus for improvement efforts. What do you think of this rationale?
  - Given your understanding of the evidence, do you think this intervention focuses on targets that will really improve outcomes?
  - Are there targets that you feel are missing?
  - Do you think there are targets proposed that are unnecessary?

**Intervention Implementation**

- How confident are you that you will be able to help advance project goals?
  - What gives you this level of confidence?
- What actions do you plan to take to ensure project success?
  - What strengths do you bring to your role as a project leader?
  - What difficulties do you foresee in advancing project goals?
  - How will you engage center staff in implementing the project?
  - Performance data will be made available to you as part of the project. Do you think that being able to see these data will affect staff’s engagement?
- Do you think that implementing this protocol will require new training for staff?
  - How do you think people will react?
- How do you feel about your center’s participation in this project?
  - How confident are you that your center will succeed in meeting targets and improving outcomes?
  - What gives you this level of confidence?
- Do you think that working together across multiple centers in this learning community will benefit the project?
