# Supplementary material for: Standardizing post-cardiac arrest care across rural–urban settings – qualitative findings on proposed post-cardiac arrest learning community intervention
Source: BMC Health Serv Res. 2023 Nov 15;23:1258. doi: 10.1186/s12913-023-10147-w (PMC10652430; doi:10.1186/s12913-023-10147-w)
Supplement: Supplementary file 2 — Additional file 2: Supplement 2. Themes and Additional Example Quotes. [file 12913_2023_10147_MOESM2_ESM.docx]

**Supplement 2: Themes and Additional Example Quotes**

**Supplement Table 2.1: Facilitators and Barriers to Current Provision of Post-Cardiac Arrest Care in System EDs**

| **Topic** | **Facilitator vs. Barrier** | **Themes and Example Quotes from P-CALC Data** |
| --- | --- | --- |
| Inner Setting | Facilitator | 2.1.1. Effective communication was cited as a strength for some ED teams:  *I think we do a really nice job with … the closed-loop communication…Everyone feels very comfortable with working with our providers…[T]hey are very open to questions and explaining and learning and teaching.... (Regional Nurse #1)* |
|  | Facilitator | 2.1.2. Teams showed strengths in provision of hyper-acute care:  *I think we do a very good job with [resuscitation]…[A]ll our nurses and everybody are trained to do [Advanced Cardiac Life Support]… (Rural Nurse #3)* |
|  | Barrier | 2.1.3. Some ED staff felt less well prepared to manage post-cardiac arrest patients for longer periods:  *I don't think [recently qualified physicians at my ED] worked in a setting [before] where they didn't have a specialist right there that they could consult [on post-cardiac arrest patients] and take over (Rural Physician #6).* |
|  | Facilitator | 2.1.4. Staff-to-patient ratios were seen as adequate at some EDs:  *[N]ot saying [our ED nurses] are not busy, but …[i]f I have a cardiac arrest patient, there will be a nurse in that room…the entire time the patient is in the department, at least one dedicated nurse, for sure (Rural Physician #2).* |
|  | Barrier | 2.1.5: Staff shortages could interfere with post-cardiac arrest management:  *[Y]ou’re trying to do multiple things…Cool the patient…place the line and intubate, start pressors, order a variety of medications. But if you don’t have enough staff to do these things…there’s certainly a delay in care (Regional Physician).* |
| System-Factors | Barrier | 2.1.6. Limited bed availability at receiving hospitals posed problems for EDs’ provision of optimal post-cardiac arrest care:  *[S]ometimes…patients at our hospital that really need a higher level of care at a tertiary care center… just don't necessarily get transferred due to… bed availability…(Regional Physician)* |
|  | Barrier | 2.1.7. Rural ED directors reported challenges negotiating administrative processes connected with post-cardiac arrest patient transfers to tertiary care centers:  *[I]t is difficult to be providing direct patient care and then be involved in that transfer process…It's hard to be the coach guiding the team when you're also on the field playing…(Rural Physician #5)* |
|  | Barrier | 2.1.8. Rural EDs reported difficulties in arranging for EMS to transport post-cardiac arrest patients to receiving hospitals:  *[A]mbulance transport is a huge, huge hurdle for us…[S]ome of these ambulance companies, they might staff a truck or two trucks and they're responsible for[local] transfers and emergencies. So they don't want to take their only truck out of town to do a transfer that's going to take an hour down to [the tertiary care center], an hour back…and any time in between prepping the patient (Rural Nurse #3).* |
|  | Barrier | 2.1.9: Tertiary care center reported receiving incomplete histories on post-cardiac arrest patients transferred from EDs at smaller system facilities:  *[W]e often feel like some of the information is getting lost in those handoffs …I always look at…what the story is with some skepticism, just because at that point it’s been translated to so many people (tertiary care center Physician).* |

**Supplement Table 2.2: Participant Attitudes toward the P-CALC Intervention**

| **Topic** | **Expressed Approval vs. Concern** | **Themes and Example Quotes from P-CALC Data** |
| --- | --- | --- |
| Intervention Goals and Principals | Approval | 2.2.1. Participants voiced support for initiatives to improve post-cardiac arrest care in EDs:  *[W]e’re learning more and more that the initial post-arrest care is really important in terms of…some of the damage that can happen after a hypoxic ischemic injury to the brain. So…there is a lot that is important to… optimizing things early on (Rural Physician #4).* |
|  | Approval | 2.2.2. Participants expressed approval for specific aspects of P-CALC intervention (standardization, targets, and rural focus):  *[A]t sites like [our rural ED]…that aren't in academic settings, it's really important to standardize processes… so you can all be practicing kind of the most recent guidelines and the highest quality of care (Rural Physician #3).*  *[A]bsolutely [these targets] will improve patient outcomes (Regional Nurse #2).*  *There's a hundred percent a disparity in care with patients in the rural settings. And… hopefully we can improve the care of this specific patient population, which is essentially the sickest patient population (Regional Physician).* |
|  | Concern | 2.2.3. Participants raised questions or concerns about aspects of the intervention rationale:  *I’ve only used paralytics once in a ROSC patient…so I was very shocked that we were using paralytics in someone who…was already intubated…I remember …going up to the pharmacy and being like, “What are we doing with this?” I’m more skeptical about it…(tertiary care center Nurse #1)*  *My understanding is that intracranial pressure is not going to go up significantly for at least a few hours after a cardiac arrest and hypoxic ischemic insult to the brain. So based on that understanding…just saying, ‘Oh, we're going to image these people once you have return of spontaneous circulation’ probably doesn't really make sense and isn't needed in every single case (Rural Physician #4).*  *[A MAP] goal of above 80 is really aggressive. And I understand the purpose of it…but in the hierarchy of importance… the temperature control and the patient's pH status really would be more important (Regional Physician).*  *[T]here’s been some question about [the TTM target]…should we try cooling or [is it] bad if we cool and then they rewarm and then we’re re-cooling again? (Rural Physician #5)* |
| Intervention Goodness-of-fit | Concern. | 2.2.4. ED services may need to be expanded because post-cardiac arrest patients now board in EDs for longer periods:  *Given recent events in medicine, we're seeing that we are sometimes being asked to take care of these [post-cardiac arrest] patients longer. So I think… we're going to be looking to…try to expand [clinical targets addressed during their ED stays] (Rural Physician #5)*  *[I]t's a small department and so we have a lot that's asked of us from many different places…[W]e might not always be able to have as much time as what we would like or what you guys would like… to put towards this [initiative] (Rural Physician #3).* |
|  | Approval | 2.2.5. System EDs need to standardize post-cardiac arrest care in order to optimize quality:  *[Y]ou can sometimes get people doing their own things that maybe aren't evidence-based or more practice-based. And I think that there's some merit to…experience, but I also do think that having a more standardized, evidence-based practice pattern that can be taught and…worked on…would probably give us… a lot of benefit (tertiary care center Physician).* |
|  | Approval | 2.2.6. Standardization of post-cardiac arrest care could help decrease stress associated with addressing a low-frequency, high-acuity event:  *[I]t's [this] sort of critical patient… that can certainly heighten anxiety among a lot of members of the team…[W]hat this [intervention] really gets to…is figuring out the early goal-directed therapies that are needed, because that's the way to help take some of … the uncertainty out of what you do in a high-acuity, but low-frequency event (Rural Physician #4).* |
|  | Approval | 2.2.7. Focus on intervention targets might impede staff’s efforts to prepare post-cardiac arrest patients for transfer:  *I think the question is…that the longer we keep [post-cardiac arrest patients] in our facilities, the issue is we're going to be possibly delaying those important interventions [provided by a tertiary care center]…(Rural Physician #6)* |
| Importance of Site-Level vs. System-Level Factors | Concern | 2.2.8: To achieve optimal post-cardiac arrest care, QI within EDs must be paired with system-level efforts to streamline transfers:  *[To attain optimal post-cardiac arrest outcomes, the health system should] not [be] allowing these patients to just be awaiting transfer for hours…(Rural Physician #3).* |
|  | Concern | 2.2.9. QI within EDs should be accompanied by standardization of communications related to transfers of post-cardiac arrest patients from EDs to tertiary care centers:  *[Standardized documentation] would just make it a lot easier as the ultimate… receiving facility to know what we're dealing with for a patient…what medications were given, how many minutes…the CPR lasted…what EMS called as the presenting rhythm, the downtimes without CPR, the bystanders, like just those things where it’s a bit more standardized and easier to find… (tertiary care center Physician)* |
| Dissemination of performance data | Approval | 2.2.10: Dissemination of system-wide and site-specific performance data on intervention targets could help support staff engagement:  *I think working on [data sharing] and showing us this patient, you improved their outcome by doing those things, will really help cement in our team’s minds, ‘Oh yeah. That [intervention] really did help a lot’(Rural Nurse #1).*  *[It is valuable for EDs to] see how other sites are doing and see if there's things that we could improve on, if we're kind of lagging behind (Regional Physician).* |
|  | Concern | 2.2.11. Physicians expressed reservations about distribution of performance data:  *[I]n the next six months, I think that data may not be very helpful. …I think we're barely treading above water in a lot of hospitals right now, as far as…the large volumes of sick patients that we're taking care of. And I think data is really helpful when you can focus on it and make changes (Rural Physician #6).* |

**Supplement Table 2.3: Facilitators and Barriers to Intervention Implementation**

| **Topic** | **Facilitator vs. Barrier** | **Themes and Example Quotes from P-CALC Data** |
| --- | --- | --- |
| Intervention Characteristics | Facilitator | 2.3.1. Intervention targets were seen as readily achievable:  *I don’t think any of these are overly ambitious. I thought they were… pretty achievable (Rural Physician #2).*  *I think [these targets are] easily addressed in a busy emergency department (tertiary care center Physician).* |
|  | Barrier | 2.3.2. Intervention focused on QI for post-cardiac arrest care is inherently challenging because patients with cardiac arrest are seen infrequently and opportunities to reinforce new practices are therefore rare:  *[C]ardiac arrest patients in general are not all that common. We see one to two a month. And…there's [an] even smaller subset where we actually have return of circulation and are looking to do the post-arrest care that we're talking about here. So it does make this more challenging because …reinforcing these things is always difficult when it's a low frequency (Rural Physician #4).* |
| Site-Level Factors | Facilitator | 2.3.3. Proposed intervention is feasible because it is compatible with ED’s current practice:  *Many of the goals are what we’re trying to do on a daily basis in the care of these patients already (Rural Physician #5).* |
|  | Barrier | 2.3.4. Low staff-to-patient ratios could result in competing demands that interfere with post-cardiac arrest-focused QI in EDs:  *[S]taffing to maintain the care of [post-cardiac arrest patients] at our facility while we're waiting for that transport can be difficult (Rural Nurse #3).* |
|  | Barrier | 2.3.5. Acute stressors like the COVID-19 pandemic could limit staff’s availability to take part in intervention implementation:  *[W]e’re…still in the middle of the pandemic…The ED nurses are being asked to do a lot of things right now. So giving them one more…guideline that they have to master possibly won’t be well-received…People are so fragile right now that one thing could set them over the edge (Rural Physician #1).* |
|  | Barrier | 2.3.6. Infrastructure limitations at rural EDs may make some targets more difficult to reach:  *We're not gonna end up doing echos at our facility…that's not something we're going to be able to do, with where we are (Rural Nurse#3).* |
|  | Barrier | 2.3.7. Staff might need new skills training to implement intervention effectively:  *We're not comfortable using paralytics in the ED, in addition to properly sedating them first… I think those two might be our biggest hurdles (tertiary care center Nurse #2).*  *[P]eople will require some training as to how to physically cool a patient, as well as some of the monitoring devices with temperature probes or arterial lines (tertiary care center Physician).* |
|  | Barrier | 2.3.8. Sites might encounter difficulties in making sure that travel nurses receive the same intervention-related training as permanent staff:  *We have a really high volume of travelers. So I think where they are so frequently in and out and here for short periods of time, making sure that the travelers we onboard are aware of the standards and protocols would be a big [challenge]…I worry about that part (Regional Nurse #2)* |
| System Level Factors | Facilitator | 2.3.9. Health system has a successful track record of implementing cross-site QI mechanisms:  *[W]e have kind of a quality review system with the system that I'm in touch with the trauma coordinators and the chief of trauma. And then same thing with STEMIs. So…there's certainly examples where we have direct kind of feedback and quality loops with the system, just not in cardiac arrest at this time (Rural Physician #3).* |
